# Fidelity to and comparative results across behavioral interventions evaluated through the RE-AIM framework: a systematic review

**DOI:** 10.1186/s13643-015-0141-0

**Published:** 2015-11-08

**Authors:** Samantha M. Harden, Bridget Gaglio, Jo Ann Shoup, Kimberlee A. Kinney, Sallie Beth Johnson, Fabiana Brito, Kacie C. A. Blackman, Jamie M. Zoellner, Jennie L. Hill, Fabio A. Almeida, Russell E. Glasgow, Paul A. Estabrooks

**Affiliations:** Human Nutrition, Foods and Exercise, Virginia Tech, Blacksburg, VA 24060 USA; Communication and Dissemination Research, Patient-Centered Outcomes Research Institute, Washington, DC 20036 USA; School of Public Affairs, University of Colorado, Denver, CO 80204 USA; Institute for Health Research, Kaiser Permanente Colorado, Denver, Colorado US; Department of Preventive Medicine, Keck School of Medicine, University of Southern California, Los Angeles, CA 90033 USA; Family and Community Medicine, Carilion Clinic, Roanoke, VA 24016 USA; Family Medicine, University of Colorado School of Medicine, Aurora, CO 80045 USA

**Keywords:** RE-AIM, Behavior change, Translation

## Abstract

**Background:**

The reach, effectiveness, adoption, implementation, and maintenance (RE-AIM) framework was developed to determine potential public health impact of interventions (i.e., programs, policy, and practice). The purpose of this systematic review was to determine (1) comparative results across accurately reported RE-AIM indicators, (2) relevant information when there remains under-reporting or misclassification of data across each dimension, (3) the degree to which authors intervened to improve outcomes related to each dimension, and (4) the number of articles reporting RE-AIM dimensions for a given study.

**Methods:**

In April 2013, a systematic search of the RE-AIM framework was completed in PubMed, PSYCHInfo, EbscoHost, Web of Science, and Scopus. Evidence was analyzed until January 2015.

**Results:**

Eighty-two interventions that included empirical data related to at least one of the RE-AIM dimensions were included in the review. Across these interventions, they reached a median sample size of 320 participants (*M* = 4894 ± 28,256). Summarizing the effectiveness indicators, we found that: the average participation rate was 45 % (±28 %), 89 % of the interventions reported positive changes in the primary outcome and 11 interventions reported broader outcomes (e.g., quality of life). As for individual-level maintenance, 11 % of studies showed effects ≥6 months post-program. Average setting and staff adoption rates were 75 % (±32 %) and 79 % (±28 %), respectively. Interventions reported being delivered as intended (82 % (±16 %)) and 22 % intervention reported adaptations to delivery. There were insufficient data to determine average maintenance at the organizational level. Data on costs associated with each dimension were infrequent and disparate: four studies reported costs of recruitment, two reported intervention costs per participant, and two reported adoption costs.

**Conclusions:**

The RE-AIM framework has been employed in a variety of populations and settings for the planning, delivery, and evaluation of behavioral interventions. This review highlights inconsistencies in the degree to which authors reported each dimension in its entirety as well as inaccuracies in reporting indicators within each dimension. Further, there are few interventions that aim to improve outcomes related to reach, adoption, implementation, and maintenance.

**Electronic supplementary material:**

The online version of this article (doi:10.1186/s13643-015-0141-0) contains supplementary material, which is available to authorized users.

## Background

The reach, effectiveness, adoption, implementation, and maintenance (RE-AIM) framework was developed to improve the balanced reporting of internal and external validities of behavioral interventions [1, 2]. This transparent and consistent reporting may lead to a better understanding of the complexity [3] and potential public health impact of behavioral interventions [1, 2]. Reach assesses the number, proportion, and characteristics of participants when compared to the target audience. Effectiveness assesses whether the targeted behavioral outcome was achieved and changes to quality of life (QOL) or other important outcomes. Adoption assesses delivery staff and setting variables (e.g., staff/setting characteristics and intervention adoption rate). Implementation assesses intervention fidelity and resources (i.e., cost and time). The maintenance dimension assesses both individual-level behavior change and organizational/setting-level intervention sustainability [1]. Accurate reporting of these dimensions enhances replication and generalizability of interventions [1]. Notably, RE-AIM includes a hyphen to differentiate the individual-level factors of reach and effectiveness from the organizational-level factors of adoption and implementation [4]. Maintenance is captured in both individual and organizational levels. Lastly, the constitutive definition of adoption includes both staff- and setting-level indicators.

The RE-AIM framework has been used to systematically review certain bodies of literature in order to make recommendations that would improve the likelihood of interventions rapidly translating from research to practice [5–8]. RE-AIM has also been used in a variety of settings such as clinics [9–11], schools [12, 13], and communities [14–16]. Furthermore, RE-AIM has been used for the planning, implementation, and evaluation of various health behavior interventions such as diabetes self-management [17, 18], weight loss interventions [19], and smoking cessation programs [20, 21]. Gaglio, Shoup, and Glasgow [22] recently completed a systematic review of studies that was based on the RE-AIM framework and found that approximately two thirds reported on all five RE-AIM dimensions. They also found that RE-AIM dimensions were not consistently operationalized and, in some cases, authors incorrectly identified and reported data for a given dimension (e.g., reported reach data as adoption [22]).

Many reviews have been conducted documenting the use of RE-AIM including small, defined bodies of literature [4, 23], broad bodies of literature [3, 24], and grant proposals [25]. To date, these reviews have primarily reported on the proportion of studies reporting on the various RE-AIM dimensions. In brief, these studies [5–8, 23] concluded that insufficient reporting of information leads to a dearth of information related to for whom, under what conditions, and how behavioral interventions are successful. Given that the Gaglio et al. review [22] identified 44 studies that reported on all RE-AIM dimensions, there is now a critical mass of articles that would allow the assessment of typically reported reach, effectiveness, adoption, implementation, and maintenance across studies.

Therefore, the purpose of this systematic review was to determine comparative results across accurately reported RE-AIM indicators, and, ultimately, to propose ways to use these findings to inform behavioral intervention work. A secondary purpose was to provide information on areas where there remains under-reporting or misclassification of data across RE-AIM dimensions. Exploratory aims included determining the degree to which authors intervened to improve outcomes related to each dimension as well as the number of articles reporting RE-AIM dimensions for a given study.

## Methods

In April 2013, a systematic literature review was completed in PubMed, PSYCHInfo, EbscoHost, Web of Science, and Scopus. The search terms were RE-AIM, RE-AIM framework, RE-AIM model, and RE-AIM methods. The date ranges were from 1999 (corresponding to the release of the seminal RE-AIM paper [1]) to April 2013. The study is not registered. To be included in the review, articles were published in English and stated the use of any of the five RE-AIM dimensions. A manuscript was excluded if categorized as a: review, commentary, theoretical paper, published abstract, dissertation, book chapter, editorial, or if it did not report on the use of RE-AIM for planning or evaluation of a study, program, or policy. Therefore, only interventions with empirical or evaluative data within the RE-AIM framework were included.

Based on the eligibility criteria (see Additional file 1 for details), three authors conducted title elimination, followed by abstract elimination. All eligible articles were assigned to pairs of investigators to independently code. Pairs of coders met to resolve discrepancies and reach consensus. The research team met for monthly progress updates and to resolve discrepancies. To determine inter-rater reliability, all members of the research team initially coded four articles. Inter-rater reliability is represented as a proportion in this manuscript.

### Data extraction

The research team consisted of scientists who previously conducted RE-AIM coding and those who had not. Each novice coder was paired with a veteran coder across all studies. Novice coders attended a training session conducted by the experienced RE-AIM investigators to ensure fidelity to the operational definitions of the extraction tool. Using an adapted extraction tool [22, 23] (see Additional file 2), the research team gathered multiple data points based on the indicators listed in Table 1. A RE-AIM abstraction tool was used rather than risk of bias assessments (i.e., those that focus primarily on randomization sequences, allocation concealment, blinding, and attrition) to ensure that the study in this manuscript reported balanced information on both internal and external validities.

**Table 1:** RE-AIM indicators by dimension

Dimension	Indicators
Reach	Method to identify target population
	Inclusion criteria
	Exclusion criteria
	Participation rate
	Representativeness
Effectiveness	Results for at least one follow-up
	Intent-to-treat analysis utilized
	Quality-of-life or potential negative outcomes
	Moderation analysis
	Percent attrition
Maintenance: individual	Assessed outcomes ≥6 months post intervention
	Qualitative measure of individual-level maintenance
	Measures of cost of maintenance
Adoption	Description of intervention location
	Description of staff who delivered intervention
	Method to identify staff who delivered intervention (target delivery agent)
	Level of expertise of delivery agent
	Inclusion/exclusion criteria of delivery agent or setting
	Adoption rate of delivery agent or setting
Implementation	Intervention duration and frequency
	Extent protocol delivered as intended
	Measures of cost of implementation
Maintenance: organizational	Indicators of program-level maintenance
	Alignment with organizational mission
	Measures of cost of maintenance

Data were gathered on the degree to which authors reported across indicators for each dimension. For dimensions that had reported indicators, outcome data were also captured. All data from articles related to a single study were combined across RE-AIM dimensions. If an indicator was misreported in one study, but appropriately addressed in another, the intervention was coded as appropriately addressing that particular indicator (this was rarely the case (*n =* 3 instances)).

### Data analysis

#### RE-AIM reporting

The findings are reported primarily as proportions and averages across studies. For reach, participation rate was calculated based on the number of participants divided by the number of members of the target population who were exposed to recruitment activities. Representativeness was assessed by describing the number of comparisons made, and differences, between the study sample and the target population or those that were eligible and declined participation. Effectiveness and individual-level maintenance outcomes were summarized based on the results of the reporting as compared to the hypothesized direction. That is, the results were coded as positive if the change to the primary outcome was in the hypothesized direction, null if there was no change from baseline, and negative if the intervention had a contrary impact on the targeted behavioral outcome (e.g., decreased physical activity, and increased rates of participants with high blood pressure).

Adoption rates were determined by dividing the number of staff/settings agreeing to deliver the intervention by the number of staff/settings that were invited to participate. Representativeness for adoption was analyzed by the number of comparisons made, and differences, between the staff/settings that agreed to participate and the staff/settings that were eligible but declined. The degree to which the intervention was implemented as intended was determined by dividing the number of intervention strategies that were implemented by the total number that were planned. A proportion calculation was to describe the number of interventions that reported making adaptations. Staff/setting-level maintenance was assessed as the proportion of staff/settings that were able to sustain the intervention over time. Finally, because providing cost and qualitative information across the RE-AIM dimensions has been encouraged, we also provide descriptions when these data were reported.

#### RE-AIM fidelity

For the secondary purpose, the proportion calculations were conducted across each dimension to determine the proportion of articles that reported on a particular indicator. If data related to a particular indicator were captured in the extraction tool, descriptive statistics were provided. At least two interventions reporting on a given indicator were required to be included in summary calculations. Therefore, if any targeted behavioral outcome (e.g., disease self-management and diet) had less than two interventions reporting on a particular item, the cell would display not applicable (N/A) within the results. Data were collected, analyzed, and synthesized until January 2015.

## Results

### Search

**Fig. 1:**
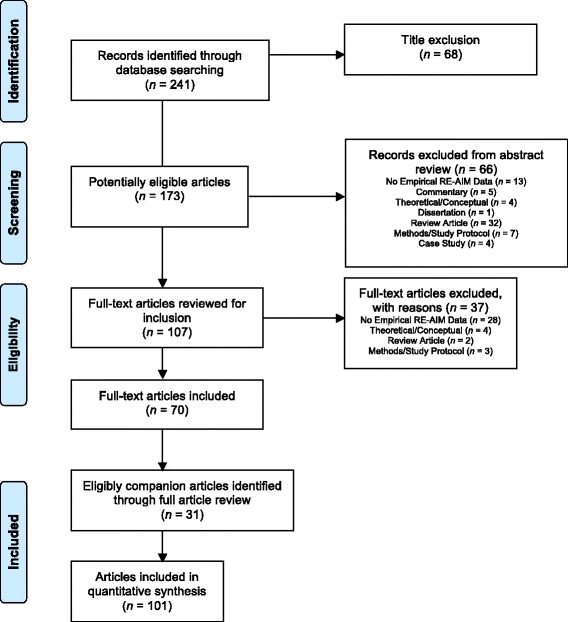
Results of literature search. PRISMA representation of search strategy and results

The original search yielded 241 potentially eligible articles. After title and abstract review, 107 articles were fully reviewed for potential inclusion and 37 were excluded. See Fig. 1 for more details. Thirty-one additional articles were referenced in eligible articles and coded as companion documents. These eligible papers (*N* = 101) represented 82 unique intervention studies for inclusion in this review. Notably, some of the original full articles assessed were based on the same intervention (i.e., companions to each other). For the remainder of the manuscript, a compilation of studies is referred to as a “trial.” See Additional file 3 for the PRISMA checklist.

### Overall summary

Inter-rater reliability was 76 % across the first four articles coded by all reviewers. The reviewers met to clarify operational definitions of codes. Across the remaining 97 articles (and 163 collected variables for each article), inter-rater reliability was over 80 %. All discrepancies were resolved.

For those trials that were represented across multiple articles (*n* = 12), there were an average of 2.58 (±1.24) with a range (*R*) of 2*–*6 articles. There was a significant difference (*p* = 0.02) in the average number of reported indicators between multiple-paper interventions (7.9 ± 3.8 indicators) and one-paper interventions (5.78 ± 2.8 indicators).

**Fig. 2:**
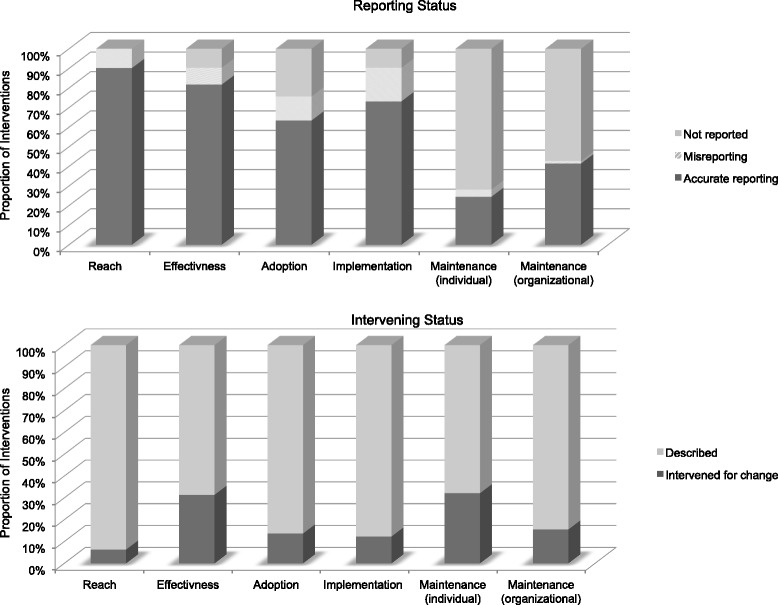
Accuracy of reporting and intervening status by dimension. This illustrates the proportion of interventions that accurately reported, misreported, or did not report on each dimension as well as the proportion of interventions that intervened to improve each dimension

Figure 2 describes (1) whether the dimension was included in the trial (i.e., reported or not reported) as well as misreported (i.e., misidentification of indicators) and (2) if the dimension was included, did the research design intervene for improved outcomes related to the said dimension or was it described for context. Related to the latter, some trials provided information describing information on a particular dimension, but the research design did not include methods to improve that particular dimension. For example, an author might describe that they approached five eligible schools to deliver an intervention (adoption), but there might not be strategies or evaluation regarding the increased uptake of the intervention at all five eligible schools. Whereas an intervention that aimed to improve the adoption rate at the school-level would include data on these efforts (e.g., attendance at relevant school meetings, identifying program champions, and provision of incentives). The most accurately reported dimension was reach (89 %), yet it was the dimension least intervened to improve (3 % of the time). All misreporting related to misidentification of individual-level variables (i.e., those that relate to the end-users) and setting-level variables.

Fifty-three percent of the trials were tested using randomized controlled trial design, 17 % were evaluation studies, 9 % were quasi-experimental, 8 % were translational/dissemination studies, 4 % were pre/post design, 3 % were cross-sectional, and 6 % were others (e.g., design included cross-sectional and observational methods). Sixty-nine percent of the studies used a quantitative methodology, 30 % were mixed methods, and one study used a qualitative approach only. Fifty-seven percent of the studies reported on the individual-level, 26 % were both at the individual- and setting-level, 14 % were at the setting-level, and 2 % accounted for individuals clustered within a setting (i.e., athletes on a team and church members within a congregation). Twenty-six trials (32 %) targeted two or more behavioral outcomes (e.g., dietary improvements *and* physical activity participation) and were operationalized as “multiple behavioral outcomes.” The remaining studies targeted smoking/substance abuse (15 %), physical activity (10 %), disease self-management (5 %), diet (5 %), weight (2 %), and other (12 %) or had no targeted individual behavioral outcome (19 %). The trials were conducted in the United States (70 %), Australia, (7 %), the Netherlands (7 %), Germany (4 %), Finland 3 %), Canada (4 %), Belgium (3 %), and one trial was conducted in both the United States and Australia. The text of this manuscript refers to the 82 trials (all articles included in the study (*N* = 101) which are summarized in Additional file 4).

### RE-AIM dimensions

The results section for each dimension describes study reporting across indicators, the outcomes that were reported, and any qualitative or cost information that was provided. Table 2 details the constitutive definition of the RE-AIM framework, while the text below provides information on each collected indicator (i.e., full employment of RE-AIM).

**Table 2:** Individual- and staff/setting-level RE-AIM dimensions by targeted behavioral outcome summary table

Dimension	Indicators	Multiple behavioral outcomes (*n* = 26)	Weight (*n* = 2)	Disease self-management (*n* = 4)	Physical activity (*n* = 8)	Diet (*n* = 4)	Smoking/substance (*n* = 12)	Others (*n* = 10)	No individual behavior outcome (*n* = 16)	Total across all behaviors (*N =* 82)
Reach	Average participation rate	49 % (±25) Studies (*n* = 18)	19 % (±12) Studies (*n* = 2)	24 % (±31) Studies (*n* = 2)	54 % (±26) Studies (*n* = 4)	N/A	52 % (±34) Studies (*n* = 6)	30 % (±18) Studies (*n* = 6)	44 % (±27) Studies (*n* = 11)	45 % (±28) Studies (*n* = 45)
	Average number of comparisons between participants and nonparticipants	4.92 (±4.07) *R* 1*–*13 Studies (*n =* 13)	N/A	N/A	3.75 (±2.50) *R* 1*–*7 Studies (*n =* 4)	2.00 (±1.41) *R* 1*–*3 Studies (*n =* 2)	5.00 (±3.00) *R* 2*–*8 Studies (*n =* 3)	2.00 (±21.73) *R* 1*–*4 Studies (*n =* 3)	1.75 (±0.95) *R* 1*–*3 Studies (*n =* 4)	4.01 (±3.53) *R* 1–13 Studies (*n* = 39)
	Average number of significant comparisons	1.63 (±0.74) *R* 1*–*3 Studies (*n =* 8)	N/A	N/A	2.00 (±1.41) *R* 1*–*3 Studies (*n =* 2)	N/A	2.50 (±2.12) *R* 1*–*4 Studies (*n =* 2)	N/A	N/A	1.05 (±1.4) *R* 0*–*13 Studies (*n* = 39)
Effectiveness	Measure of primary outcome	Positive (*n* = 20) null (*n* = 3) misreport (*n* = 0) not reported (*n* = 3)	Positive (*n* = 2) null (*n* = 0) misreport (*n* = 0) not reported (*n* = 0)	Positive (*n* = 2) null (*n* = 0) misreport (*n* = 0) not reported (*n* = 2)	Positive (*n* = 6) null (*n* = 1) misreport (*n* = 1) not reported (*n* = 0)	Positive (*n* = 4) null (*n* = 0) misreport (*n* = 0) not reported (*n* = 0)	Positive (*n* = 8) null (*n* = 1) misreport (*n* = 1) not reported (*n* = 2)	Positive (*n* = 6) null (*n* = 1) misreport (*n* = 3) not reported (*n* = 0)	N/A (*n* = 8) misreport (*n* = 8)	Positive (*n* =48) null (*n* = 6) misreport (*n* = 13) not reported (*n* = 7) N/A (*n* = 8)
Maintenance^a^	Measure of primary outcome ≥6 months post-treatment	Improved outcome from baseline to follow-up (*n* = 6) not reported (*n* = 20)	Improved outcome from baseline to follow-up (*n* = 0) not reported (*n* = 2)	Improved outcome from baseline to follow-up (*n* = 0) not reported (*n* = 4)	Improved outcome from baseline to follow-up (*n* = 1) not reported (*n* = 7)	Improved outcome from baseline to follow-up (*n* = 0) not reported (*n* = 4)	Improved outcome from baseline to follow-up (*n* = 2) not reported (*n* = 10)	Improved outcome from baseline to follow-up (*n* = 0) not reported (*n* = 10)	Improved outcome from baseline to follow-up (*n* = 0) not reported (*n* = 16)	Improved outcome from baseline to follow-up (*n* = 9) not reported (*n* = 73)
Adoption	Average percentage of settings approached that participate	73 % (±35) Studies (*n =* 12)	N/A	93 % (±10) Studies (*n =* 2)	65 % (±37) Studies (*n =* 5)	N/A	68 % (±33) Studies (*n =* 3)	95 % (±7) Studies (*n =* 5)	56 % (±40) Studies (*n =* 5)	75 % (±32) (*n* = 33)
	Average number of comparisons between participating sites compared with nonparticipating	2.60 (±2.19) *R* 1*–*5 Studies (*n* = 5)	N/A	N/A	2.50 (±2.07) *R* 1*–*6 Studies (*n* = 6)	N/A	N/A	1.00 (±0.00) Studies (*n* = 2)	1.00 (±0.00) Studies (*n* = 3)	0.56 (±0.98) Studies (*n* = 32)
	Average number of significant comparisons	1.0 (± .25) *R* 0*–*5 Studies (*n* = 4)	N/A	N/A	N/A	N/A	N/A	N/A	1.00 (±0.00) Studies (*n* = 2)	0.32 (±0.58) Studies (*n* = 20)
	Percent of staff offered that participate	76 % (±32) Studies (*n =* 5)	N/A	N/A	85 % (±22) Studies (*n =* 3)	N/A	N/A	95 % (±7) Studies (*n =* 3)	85 % (±15) Studies (*n =* 3)	79 % (±28) Studies (*n* = 12)
	Characteristics of staff participants vs nonparticipating staff or typical staff	2.50 (±2.12) *R* 1*–*3 Studies (*n* = 2)	N/A	N/A	N/A	N/A	N/A	N/A	1.00 (±0.00) Studies (*n* = 5)	1.92 (±1.68) Studies (*n* = 12)
	Average number of significant comparisons	N/A	N/A	N/A	N/A	N/A	N/A	N/A	N/A	0.08 (±0.29) Studies (*n* = 12)
Implementation	Measure of implementation fidelity	Description (*n =* 5) Percentage (*n =* 10) *M =* 87 % (±17)	Description (*n =* 1) Percentage (*n =* 1)	Description (*n =* 1)	Description (*n =* 2) Percentage (*n =* 4) *M =* 71 % (±12.23)	Percentage (*n =* 1)	Description (*n =* 1) Percentage (*n =* 3) *M =* 84 % (±10.40)	Description (*n =* 1) Percentage (*n =* 2) *M =* 76 % (±33.23)	Description (*n =* 5)	Those that described (*n =* 21) Average percentage 82 % (±16)
	Cost of implementation—money	*n =* 2 (1) $547 per person, (2) “low cost”	*n =* 1 Lay health educators and free program materials	*n =* 0	*n =* 0	*n =* 0	*n =* 0	*n =* 0	*n =* 3 (1) $6.91/person, (2) low ongoing costs, (3) 266,000 Euros for 3 years	Costs reported in six studies
Maintenance^b^	Reported if program is still ongoing at ≥6 months post-treatment	Studies (*n =* 1)	Studies (*n =* 1)	Studies (*n =* 0)	Studies (*n =* 5)	Studies (*n =* 3)	Studies (*n =* 1)	Studies (*n =* 3)	Studies (*n =* 7)	Studies (*n =* 27)

*N/A* Not applicable

^a^Organizational

^b^Individual

### Individual-level outcomes

#### Reach

Overall, 17 % of the trials reported on all four indicators of reach (see Table 2). Those that reported a method to identify the target population (*n* = 50) used existing records (e.g., medical and registry). Sixty-eight percent of the trials reported at least one eligibility criterion, and of those, 25 explicitly stated exclusion criteria. These eligibility criteria were typically related to: age (*n* = 37), membership (*n* = 33; e.g., church and school), physical or mental condition (*n* = 14), language (*n* = 14), tobacco use (*n* = 11), location (*n* = 9), activity level (*n* = 9), access to phone (*n* = 4), and others (*n* = 3: gender, lost job, and completed screening). The participation rate was accurately reported for 55 % of the trials, 10 % of the trials misreported participation rates, and one trial accurately reported reach in some articles but not others.

The median number of participants was 320 (mean (*M*) = 4817 (±28,656); *R* 28*–*234,442). The trials that accurately reported on the participation rate were able to reach 45 % (±28) of eligible and invited individuals, with a range from 2 to 100 %. Thirty-seven trials (48 %) reported on representativeness. The number of characteristics compared ranged from 1 to 13 with a mean of 3.90 (±3.30). Of those that examined representativeness, 17 (46 %) found at least one significant difference between those that participated and the target population; the most common characteristics were that participants in these behavioral trials were more often of Caucasian race (*n* = 5), of higher income (*n* = 3), and of higher education (*n* = 2). There were also seven studies that found significant differences in age between participants and nonparticipants; some were older than the target audience (*n* = 4) and others were younger than the target audience (*n* = 3). All other characteristic comparisons were only reported as a significant difference in one trial (e.g., profession, comorbidities, and English language).

Four trials (9 %) included qualitative data to address reach. One telephone interview protocol evaluated the reach of program awareness, in which they found that 35 % of eligible residents responding were aware of the program [26]. In a hospital worksite obesity prevention trial [27, 28], researchers captured open-ended responses for the reasons eligible persons declined participation and found reasons to include lack of interest (56 %), no time (19 %), and personal health or family obligations (2 %) while 22 % gave no reason. For one trial, interviewees from ten focus groups described barriers and facilitators of participation in a worksite smoking cessation intervention [29, 30]. Respondents provided data related to the recruitment methods to which they were exposed and reported that better marketing, supervisor encouragement, weekly bulletins, and announcements at worksite meetings would increase participation [29, 30]. Four trials also reported on the costs of recruitment. Of those, three reported numerical values (R$10–252.54 per participant [31–40]), while one study reported information that could be used to determine recruitment costs (e.g., the costs associated with interactive voice response system that made 40,185 calls across 3695 individuals [41]).

#### Effectiveness

One trial [42–45] accurately reported on all five indicators within this dimension. Of those that accurately reported effectiveness on individual behavior outcomes (*n* = 55), 89 % had positive findings on the behavioral outcome and 11 % had null findings. These results are presented by targeted outcome in Table 2.

Twenty-five percent of the trials (*n* = 19) included a moderation analysis to determine robustness across subgroups. Eleven trials (14 %) reported broader outcomes, QOL, or unintended negative outcomes. Some measures included the Centers for Disease Control and Prevention’s Healthy Days measure [46], Patient Health Questionnaire (PHQ) [46, 47], and Problem Areas in Diabetes 2 (PAID-2) scale [47]. Five trials used qualitative measures of effectiveness; three of which used open-ended survey items and two conducted interviews. Twenty-one trials reported attrition rates (*M* = 22 %). Qualitative data related to effectiveness primarily focused on participant experiences [29, 30, 41, 42, 48, 49] and suggested that program adaptations for specific sub-populations could improve participant perceptions of effectiveness [47]. Only three trials reported any measure of the costs associated with effectiveness: two reported costs per participant ($4634 and $1295 [33–40]) and the other one reported that costs were considered in the design and analysis [51].

#### Individual-level maintenance

None of the studies reported on all three indicators of individual-level maintenance. However, nine trials (11 %) reported individual-level behavior change at least 6 months post-treatment. All nine reported positive outcomes when compared to baseline. One study included qualitative interviews through which participants indicated the need for stronger volunteer and staff support to bolster individual-level maintenance [52]. None of the studies reported individual-level maintenance costs.

### Setting-level outcomes

#### Adoption

One trial, across two studies [31, 32], reported on all six indicators of adoption. Sixty-three percent of the trials (*n* = 52) reported on both staff- and setting-level adoption factors. Forty percent of the trials reported setting-level adoption rates, which was, on average, 75 % (±32). Fifteen of the trials (19 %) reported setting-level eligibility criteria; these criteria included size, location, demonstration of need, and being within a particular health insurance network. Twenty trials (26 %) compared the characteristics of participating settings to all targeted settings. Five trials found significantly different characteristics, which included: single-physician practices being less likely to participate, governmental sector being more likely to participate, and those who had an increase in the number of patients/respondents over time were more likely to participate.

The average staff-level adoption rate was 79 % (±28). Sixteen studies (20 %) reported delivery agent eligibility (i.e., criteria that enables an individual to deliver the intervention (e.g., education and role within the system)). These criteria were usually based on expertise (*n* = 6), affiliation with targeted setting (*n* = 4), and other disparate criteria such as not planning on retiring or having enough patients. Ten trials (12 %) compared the characteristics of participating settings to all targeted settings (*M* = 1.30 comparisons (±0.9); *R =* 1–4). Only one study found significant comparisons of participating staff to eligible staff. In this case, the delivery staff was more likely to be women and reported more years of experience in physical activity program delivery [26]. All setting and staff indicators can be found in Table 2.

Thirteen studies used qualitative measures for adoption and found that adoption rates were improved through partnerships and increased awareness. For example, Vick et al. [53] found that the lack of awareness, combined with scheduling conflicts, decreased the likelihood of staff attending training; whereas partnering with representatives within the organization led to strategic, feasible, and well-accepted training sessions and intervention [54]. Only two studies reported monetary values associated with adoption. One reported a total adoption cost of $21,134 [35–40] while the other indicated $15 per hour to train coaches [55].

#### Implementation

One study reported all three implementation indicators [21]. Thirty-five trials (44 %) reported on the degree to which the program was delivered as intended. Across all targeted outcomes, the average percent fidelity was 81 % (±16.49). Seventeen trials (22 %) reported that adaptations were made to program delivery. Thirty trials (39 %) provided information on the number and frequency of trial contacts, which represented the resource of “time.” Eighteen of the trials (24 %) used qualitative inquiry for implementation: surveys (*n* = 7), interviews (*n* = 6), observations and interviews (*n* = 2), focus groups (*n* = 2), and an implementation checklist (*n* = 1). Qualitative inquiry identified barriers and facilitators of implementation. Example barriers included scheduling and staff turnover [56] as well as a lack of role clarity (i.e., understanding ones responsibilities related to the intervention) [57] while successes were attributed to increased patient trust of care providers [49] and multilevel commitment (e.g., management and investment of partnerships [57]). Eight percent of the trials (*n* = 6) reported at least some data around implementation monetary costs (e.g., program updates and manuals) but did not include raw data on costs.

#### Organizational-level maintenance

None of the studies reported on all three indicators within maintenance. Eleven of the trials (13 %) reported alignment with an organizational mission. Twenty-eight of the trials (34 %) reported on whether or not the program was still in place. Of those that reported on institutionalization of the program, 16 (62 %) were still in place. Eleven trials (13 %) included information on modifications that were made for system-level maintenance. Seven trials reported on organizational attrition (*M* = 9.82 % (±10.55)). Finally, 15 % reported qualitative measures of maintenance via interviews (*n* = 10) and open-ended surveys (*n* = 2). These data indicated compatibility with their delivery system and delivery agent skill set as well as a wide array of themes from ongoing staff and management support (support of duration, frequency, and type of trial). No salient barriers were identified via the interviews and open-ended surveys. No data were reported on costs of organizational-level maintenance.

## Discussion

The purpose of this review was to move beyond an assessment of the adequacy of reporting across the dimensions of the RE-AIM framework to include outcome data related to each dimension. A number of conclusions are made from this review to provide directions for future research.

First, at the individual-level, participation rates were varied across behavioral target. Regardless of sample size, though, the vast majority of trials had a positive impact on effectiveness. Studies testing interventions targeting multiple behavioral outcomes appear to attract participants at a higher rate than those that focus on weight management. For example, there were 234,442 participants in a statewide evaluation of school-aged youth’s physical activity and dietary behaviors [58]. Similarly, a targeted weight management and physical activity trial had 1952 participants [59]. However, the study with only 28 participants [56] was a specific alcohol referral program. Reasons for these differences in sample sizes are also connected to setting-level factors (e.g., state-, school-, community-, or clinic-wide interventions versus pilot testing) and demonstrate the need to scale interventions to have a broad public health impact.

Second, over half of the studies that report on representativeness found that their sample was generalizable to the target population. Thirty-nine studies included a comparison between the recruited participants and the target audience. One study [60] found that participants were significantly more likely to be Caucasian and older than the target audience. In contrast, other researchers found that participants in a diabetes prevention program for older adults were younger than the target audience [61, 62]. In general, those that found their samples were less representative, the typical differences included an over representation of Caucasians and those with higher income and education levels. In an effort to move toward health equity [63], researchers need to be persistent in targeting and recruiting participants from minority communities, those of low income, and those of lower education.

Third, adoption and implementation rates were relatively high for settings and staff that agree to deliver a given intervention, though data on representativeness at these levels were scarce. This may indicate that studies with more positive results were more likely to include these fidelity calculations in their articles. There is a gap in the literature related to personal characteristics and perceptions of the intervention from those who deliver interventions [64]. That is, the expertise reported often alluded to “trained” delivery agents without providing details about how a “trained” delivery agent was defined. Many studies reported some degree of intervention fidelity, although very few reported an actual percentage of intervention content that was delivered as intended. Only one study included an implementation checklist to systematically document the delivery of intervention components [49]. This lack of data related to staff/setting-level factors may hinder intervention adoption and sustainability.

Fourth, the majority of trials that employ the RE-AIM framework test interventions that target effectiveness (~60 %) and very few target reach or organizational maintenance (~5 % each). While the RE-AIM framework was developed to address outcomes across each dimension, there were relatively few studies that examine reach or staff/setting-level dimensions as the target for intervention. A key principle from the RE-AIM perspective is that a public health impact can be improved by maximizing outcomes for each dimension [1]. Future work on RE-AIM would benefit from interventions that systematically plan to test ways to improve reach, adoption, implementation, and setting-level maintenance. To do this, researchers must plan intervention design, delivery, and evaluation with the real-world application in mind. In fact, the Behavior Change Consortium developed a systematic way in which to accomplish this [63]. Essentially, interventionists can use the indicators (as seen in Table 1) to develop, deliver, and evaluate an intervention. By addressing these complex issues in the planning stages, researchers can more readily understand the potential public health impact of a proposed intervention [3].

Fifth, our results provide information on areas where there remains under-reporting or misclassification of data across RE-AIM dimensions. Authors often reported an inaccurate denominator within the dimensions of reach and adoption. That is, within reach and adoption, “those who decline” or “were unable to be contacted” should not be categorized as ineligible by default. For these reasons, we suggest using multiple indicators to accurately communicate the number, proportion, and representativeness of participants, settings, and staff [65]. Cost was also rarely and inconsistently reported. Consistent reporting of reach and adoption would further the field of implementation science in that it would highlight the types of people and settings that are not being recruited into interventions and lead to a concerted effort to improve these rates by tailoring intervention materials and approaches.

We also found that some reports of indicators were more vague (e.g., cost and quality of life) than others, making it difficult to discern comparisons across targeted behavioral outcomes. However, we recognize that there are often practical limitations (with word and space limitations of journals) to thoroughly include all indicators related to each dimension. For this reason, we suggest (1) reporting dimensions across multiple papers, as needed, and (2) using tabular representations with headers such as “Dimension, Outcome, Measures, and Results” to be clear and consistent. Notably, related to our exploratory aim of determining the number of articles used by authors to report across RE-AIM dimensions, only 12 of the trials were reported across multiple papers, and those trials that were reported across multiple papers included more RE-AIM indicators than trials reported in a single manuscript. This provides preliminary support for reporting dimensions across multiple papers when a full RE-AIM analysis is not feasible for the targeted journal.

As this was the first study to provide preliminary evidence related to comparative results across each dimension, there was notable variation across both the degree to which indicators were included and our ability to make specific inferences. That is, there were not enough studies that accurately reported data to categorize low, moderate, and high reach, adoption, implementation, and maintenance rates. However, we presented comparative results where possible as well as provided salient reporting issues and suggestions for improvements. Secondly, the present study does not evaluate variation in operational definitions of the indicators posed by the authors of the trials. Notably, the RE-AIM indicators remain the same regardless of intervention type, target audience, settings, etc. We included information on intervention type, evaluation metrics, level of evaluation (e.g., individual and setting), and the degree to which interventions intervened to improve a particular dimension. For consistency, trained coders reported indicators as “accurate,” “misreported,” or “not reported” according to the constitutive definitions of RE-AIM [2].

## Conclusions

The RE-AIM framework has been employed in a variety of populations and settings and for the planning, delivery, and evaluation of behavioral interventions. The RE-AIM framework was developed to place equal importance on all five dimensions of interest in order to translate behavioral interventions into sustained practice and have a large public health impact [1]. Yet, this review highlights that there are still inconsistencies in the degree to which authors are reporting each dimension in its entirety as well as inaccuracies in reporting indicators within each dimension. Further, there are few interventions that aim to improve outcomes related to reach, adoption, implementation, and maintenance. Taken together, this review points to a pipeline for future research: increased accuracy and transparency across all five dimensions to enhance replication, generalizability, and translation as well as the need to intervene to improve outcomes within each dimension.

## Additional files

Additional file 1: Eligibility criteria. This table presents the eligibility criteria for the systematic review. (PDF 76 kb) Additional file 2: Data extraction tool. This table reflects the data extraction tool with each of the RE-AIM indicators and their operationaldefinitions for coders. (PDF 113 kb) Additional file 3: PRISMA checklist. This file adheres to the Preferred Reporting Items for Systematic Reviews and Meta-Analyses(PRISMA) checklist to improve transparency of the methods and results of this systematic review. (PDF 116 kb) Additional file 4: Summary of all articles included in the review. As manuscripts were grouped together to reflect interventions, this table provides summary data on all individual articles included in the review. (PDF 417 kb)

## Abbreviations

PHQ: Patient Health Questionnaire
QOL: quality of Life
