# Supplementary material for: Fidelity to and comparative results across behavioral interventions evaluated through the RE-AIM framework: a systematic review
Source: Syst Rev. 2015 Nov 8;4:155. doi: 10.1186/s13643-015-0141-0 (PMC4637141; doi:10.1186/s13643-015-0141-0)
Supplement: Additional file 1: Eligibility criteria. — This table presents the eligibility criteria for the systematic review. (PDF 76 kb) [file 13643_2015_141_MOESM1_ESM.pdf]

Additional file 1. Eligibility criteria for articles.

**Data type**	**Inclusion criteria**
RE-AIM	· State the use of any RE-AIM dimension<br>· Use RE-AIM in empirical and evaluation purposes
Language	· English
Study design	· Used experimental or quasi-experimental design
Assessments	· Must include data on at least one RE-AIM dimension
Date ranges	· 1999 to April 2013
**Excluded**	· Commentary<br>· Theoretical papers<br>· Published abstracts<br>· Dissertations<br>· Book chapters<br>· Editorials<br>· Reviews
