# Supplementary material for: Fidelity to and comparative results across behavioral interventions evaluated through the RE-AIM framework: a systematic review
Source: Syst Rev. 2015 Nov 8;4:155. doi: 10.1186/s13643-015-0141-0 (PMC4637141; doi:10.1186/s13643-015-0141-0)
Supplement: Additional file 2: Data extraction tool. — This table reflects the data extraction tool with each of the RE-AIM indicators and their operationaldefinitions for coders. (PDF 113 kb) [file 13643_2015_141_MOESM2_ESM.pdf]

**Additional file 2.** RE-AIM extraction tool.	
**Article ID Number**	**Located in the assignment sheet**
Author, Year, Country	
Target Population	Brief description of the targeted population (older adults, pre-diabetic women)
Study Setting	The location(s) where the intervention is delivered. Example: university/clinical/community/faith-based/home/worksite
Topic Area	List the health behavior or health topic that is covered (such as cancer, policy, diabetes…).
Purpose	What is the purpose of the manuscript? Provide details that will give us highlights of the paper (e.g., the purpose of this paper was to report on the short term effectiveness of a dietary program, with special attention on the feasibility (adoption) of the program in a clinical setting).
Study Design	RCT, CC, Observational, etc.
Methods used:	Quantitative, qualitative, review article, meta analysis, narrative piece…
Level/Unit of Analysis:	Individual, setting, community, combination...
Companion Article	Is there a companion article to this particular intervention (yes = 1; no =0)?
Citation	If so, what is the citation we will need to search (later)?
REAIM Overview Table *	O= organizational I= individual
Assessed?	The assessed column is simply a description of if this dimension is addressed (yes = 1; no =0).
Change or Description or Neither	For this column indicate if the intervention attempted to change the outcome related to this dimension; described the outcome; or did neither
Measure	This is the specific information on how the outcome was assessed.
Challenge/Finding?	Description of any finding based on the study or if there were challenges recorded by the authors.
Reach	The proportion & representativeness of individuals willing to participate in a given intervention
Described target population	A brief description of the broader target population (i.e., not simply of the study sample). Example: The target population included all women within the community health center who were over the age of 18 and were not meeting the recommended guidelines for physical activity.
Demographic & behavioral information	Gender, age, educational attainment, occupation, SES, behavioral outcomes

Method to identify target population	Describe the process by which the target population was identified for participation in the study.<br>Example: All patients who were part of the target population were identified using the electronic medical record.
Recruitment Strategies	Describe the methods used to recruit participants into the study.<br>Example: We used a series of flyers; presentations; mass media; and word of mouth strategies to recruit participants.
Inclusion criteria	Explicit statement of characteristics of the target population that were used to determine if a potential participant is eligible to participate.<br>Example: The inclusion criteria are…
Exclusion criteria	Explicit statement of characteristics that would prevent a potential participant from being eligible to participate. Also the percent excluded may be reported.<br>Example: The exclusion criteria are…
Number eligible and invited(exposed) to recruitment	The total number of eligible participants contacted for participation.<br>Example: 300 people were contacted for the study. After a screener was administered, it was found that of those 300 people contacted, 250 people were eligible. Therefore 250 is the denominator.
Sample size	The number of people who agree to participate (e.g., n=).
Participation rate	Sample size divided by the target population denominator<br>Example: 200 (number of people agree to participate)/250 (number of eligible participants contacted for participation)=80%
Number of Characteristics	Total number of comparisons made between target population and study sample with a description of what those comparisons were.<br>Example: Participants were compared to non-participants for: activity level, gender, age...
Number of statistically significant comparisons	Total number of statistically significant comparisons made between target population and study sample with a description of what those comparisons were.<br>Example: When compared to participants, non-participants were more likely to be older physically inactive females.
Cost of recruitment	The cost of recruitment can reflect monetary and/or time units.<br>Example: The overall cost of recruitment strategy A (flyers) was $1000 versus the overall cost of recruitment strategy B (newspaper advertisements) was $200. Could also be coded in cost per participant recruited.
Use of qualitative methods to measure reach	Reporting on non-quantitative aspects of reach. Observations in words, sentences, descriptions or codes; Some common methods include key informant interviews, focus groups, or even field notes that provide information on perceptions, feelings, opinions, experiences, etc.
Efficacy/Effectiveness	The influence of an intervention on important outcomes, including potential negative effects, quality of life, & economic outcomes
Report of Mediators	Variables that explain the extent to which the particular variable accounts for the relationship between the predictor and the criterion.

Report of Moderators	List of variables: qualitative (e.g., sex, race, class) or quantitative (e.g., level of reward) that influence the direction and/or strength of the relationship between the treatment and the outcome.
Intent-to-treat or present at FU?	Intent to treat analysis: When participants in trials are analyzed in the groups to which they were randomized, regardless of whether they received or adhered to the allocated intervention. Example, will typically use the term intent to treat or will describe an imputation that was used to account for missing data in the analysis.<br>Present at Follow-up analysis: When only participants who completed the follow-up assessment are included in the analysis of efficacy/effectiveness.<br>Example: Only those participants who completed both the baseline and follow-up measures were included in the analysis.
Imputation procedures (specify)	Substitution of some value for missing data.<br>Example: Multiple imputation methods were used to impute missing minutes of PA data at 3 months…
Quality of life measure	Includes a measure of quality of life with some latitude for coding articles that refer to well-being or satisfaction with life.
Measure unintended consequences (negative) and results	To evaluate unanticipated consequences and results that may be a product of the intervention and may have caused unintended harm.<br>Example: In a PA promotion program, female participants had an increased rate of injury.
Percent attrition (at program completion)	The proportion that was lost to follow-up or dropped out of the intervention. This is calculated by dividing the number of participants who did not complete the intervention by the number of participants who began the intervention.<br>Example: 100 participants began the intervention and 20 participants did not complete the intervention. So there was 20% attrition.
Cost effectiveness	Code as reported if specific mention and amounts are provided for the cost of the intervention.<br>Example: The new strategy would save $1,000 per life per year when compared to the current practice.
Use of qualitative methods to measure efficacy/effectiveness	Obtaining qualitative feedback from participants on the degree to which they felt the intervention was efficacious/effective. Some common methods include focus groups, interviews, diaries (text/pictures).
Adoption - Diffusion - Setting Level	The proportion & representativeness of locations willing to initiate & adopt an intervention
Number eligible and invited(exposed)	Total sites that met eligibility criteria and were approached for intervention delivery.
Number participating	The total number of sites that agreed to participate.
Participation rate	The proportion of sites eligible and contacted that participated.
Description of targeted location	Characteristics that would be considered an ideal location for the intervention.

Inclusion/exclusion criteria of setting	The explicit statement of characteristics of the setting that were used to determine if a potential setting is eligible to participate.<br>Example: The inclusion/exclusion criteria are...
Description of intervention location	The explicit statement of characteristics of the location of the intervention.<br>Example: size of location; resources available staff information; number of eligible locations; work environment/climate
Method to identify setting	Describe the process by which the location was identified for participation in the study.
Number of Comparisons	Total number and type of comparisons of targeted intervention sites and those that participated, including a list: size, location, etc.
Number of statistically significant comparisons	Total statistically significant number and type of comparisons of targeted intervention sites and those that participated, including a list: Schools in urban areas were significantly less likely to agree to participate.
Average number of persons served per setting	Calculated average number of participants at each site.
Adoption - Diffusion - Staff Level	The proportion & representativeness intervention staff willing to initiate & adopt an intervention
Number eligible and invited(exposed)	Total staff that met eligibility criteria and were approached for intervention delivery.
Number participating in delivery	The total staff members that agreed to participate.
Participation rate	The proportion of the staff that was eligible and contacted and participated.
Method to identify target delivery agent	Describe the process by which the target delivery agent was identified for participation in the study.<br>Example: All staff at the intervention location that had expertise in leading PA classes was identified by supervisors at the intervention location.
Level of expertise of delivery agent	Training or educational background in relevant area; Degrees, certifications of delivery agents (such as PhD, Masters, Registered Dietitian, etc.)
Inclusion/exclusion criteria of delivery agent	The explicit statement of characteristics of the delivery agents that were used to determine if a potential delivery agent is eligible to participate.<br>Example: The inclusion/exclusion criteria are...
Number of comparisons	Total number and type of comparisons of targeted staff members and those that participated, including a list: age, BMI, education
Number of statistically significant comparisons	Total statistically significant number and type of comparisons of targeted intervention sites and those that participated, including a list: Staff members who were overweight were less likely to agree to deliver the program.
Measures of cost of adoption	The price of adoption across all levels of the intervention; At least some mention of start-up (i.e., **not ongoing** just **one time** (start-up)) costs

Dissemination beyond originally planned	The spread of the intervention beyond what was planned before the start of the intervention.
Use of qualitative methods to measure adoption	Used qualitative methods to understand the process of adoption.<br>Example: focus groups, interviews of adoption settings or delivery agents
Implementation	How consistently various elements of an intervention are delivered as intended by intervention staff, & the time & cost of the intervention
Theories	Explicit statement of theories or principles used to develop the intervention<br>Example: social cognitive theory, theory of planned behavior, social ecological model, dissemination & implementation-based theories
Intervention number of contacts	Total number of encounters with participants; Could include face-to-face meetings, telephone calls, newsletters etc.
Timing of contacts	Describe when the intervention contacts occur over the course of the intervention.<br>Example: For the first month participants received one telephone call per week and in every month thereafter they received a call a month until the end of the 12 month intervention
Duration of contacts	Length of each intervention contact<br>Example: The first 4 calls lasted about 20 minutes each, the other 11 lasted about 10 minutes each.
Extent protocol delivered as intended	Description of fidelity to the intervention protocol<br>Example: a checklist of program components assessed by delivery agent(s)
Consistency of implementation across setting and delivery agents	Description of the degree of similarities between multiple settings sites & delivery agents
Participant attendance/completion rates	The proportion of the intervention that the participants received, on average.<br>Example: Participants attended 4 of the 6 meetings on average.
Measure of cost	The **ongoing** cost of delivery across all levels of the intervention
Use of qualitative methods to measure implementation	Used qualitative methods to understand the process of implementation.<br>Example: focus groups, interviews
Maintenance	The extent to which participants make & maintain a behavior change & the sustainability of a program or policy in the setting in which it was intervened
Was individual behavior assessed at some duration following the completion of the intervention? (give duration of follow-up)	Description of follow-up outcome measures of individuals available at some duration after intervention termination<br>Example: 6 months after the intervention ended participants had returned to baseline levels of PA.
Attrition	Describe the degree to which participants were lost to follow-up (and the reasons) during the period in time from the interventions completion to the follow-up.

Use of qualitative methods to measure individual maintenance	Used qualitative methods to understand the process of individual level maintenance of changes to the primary outcome.<br>Example: focus groups, interviews
Report alignment to organization mission	Was the intervention designed to align with the delivery organization's mission, values (explicit statement)?
Is the program still in place?	Description of program continuation after completion of the research study.
If no: reason for discontinuation	Description of why the intervention was terminated.
If yes: was the program modified? Specify	Description of any changes that were made to the original program.
Was the program institutionalized?	Description of the how the intervention was integrated into the delivery system through methods such as policy changes, job description changes.
Attrition	Describe the degree to which sites were lost to follow-up (and the reasons) during the period in time from the interventions completion to the follow-up.
Use of qualitative methods to measure organizational level maintenance	Used qualitative methods to understand the process of intervention sustainability at the organizational level.
Comparative Results	
Weight Measure	Yes or No
Disease self-management	Yes or No
Quality of Life Measures	Yes or No
Physical Activity	Yes or No
Dietary	Yes or No
Smoking/Substance	Yes or No
Diabetes	Yes or No
Other?	Yes- Please provide
