# Supplementary material for: Fidelity to and comparative results across behavioral interventions evaluated through the RE-AIM framework: a systematic review
Source: Syst Rev. 2015 Nov 8;4:155. doi: 10.1186/s13643-015-0141-0 (PMC4637141; doi:10.1186/s13643-015-0141-0)
Supplement: Additional file 4: Summary of all articles included in the review. — As manuscripts were grouped together to reflect interventions, this table provides summary data on all individual articles included in the review. (PDF 417 kb) [file 13643_2015_141_MOESM4_ESM.pdf]

Additional file 4 . Summary of all included RE-AIM studies (*N*=101)

Title	First Author	Year	Country	Target Population	Study Setting	Study Design	Methods Used	Level of Analysis	Reach Assessed? *	Reach Intervened? +	Effectiveness Assessed? *	Effectiveness Intervened? +	Adoption Assessed? *	Adoption Intervened? +	Implementation Assessed? *	Implementation Intervened? +	Maintenance(I)_Assessed? *	Maintenance(I)_Intervened? +	Maintenance(O)_Assessed? *	Maintenance(O)_Intervened? +	Outcome
A 3-month jump-landing training program: A feasibility study using the RE-AIM framework	Aerts	2013	Belgium	Basketball players in Belgium	Second highest national level and regional basketball leagues	RCT	Mixed Methods	IWS	Y	D	Y	C	Y	D	Y	D	N	N	Y	D	Injury prevention
Promoting fruit and vegetable consumption among members of black churches, Michigan and North Carolina, 2008-2010	Allicock	2013	U.S.	African American churches/ church members	North Carolina and Michigan churches	RCT	Mixed Methods	IWS	Y	D	Y	C	Y	D	Y	D	Y	D	Y	D	Dietary
Evaluation of a pilot training program in alcohol screening, brief intervention, and referral to treatment for nurses in inpatient settings	Broyles	2013	U.S.	Nurses at a VA hospital	Large academic medical center affiliated with the US. Department of Veterans Affairs	RCT	Mixed Methods	I	Y	D	Y	D	Y	D	Y	D	N	N	Y	D	Substance abuse
Sustained use of a tool for lifestyle intervention implemented in primary health care: a 2-year follow-up.	Carlfjord	2013	Sweden	Providers in Swedish primary care settings	Swedish primary health care settings	Translational	Quantitative	S	Y	D	Y	C	Y	C	Y	D	Y	D	N	N	No individual outcome

Appendix D. Summary of all included RE-AIM studies (*N*=101)

Title	First Author	Year	Country	Target Population	Study Setting	Study Design	Methods Used	Level of Analysis	Reach Assessed? *	Reach Intervened? +	Effectiveness Assessed? *	Effectiveness Intervened? +	Adoption Assessed? *	Adoption Intervened? +	Implementation Assessed? *	Implementation Intervened? +	Maintenance(I)_Assessed? *	Maintenance(I)_Intervened? +	Maintenance(O)_Assessed? *	Maintenance(O)_Intervened? +	Outcome
Evaluation of a theory-driven e-learning intervention for future oral healthcare providers on secondary prevention of disordered eating behaviors.	Debate	2013	U.S.	Dental students	Dental hygiene and dental schools	Effectiveness study	Quantitative	I	Y	D	Y	D	N	N	N	N	N	N	N	N	No individual outcomes
N'Balance: A community-based fall-prevention intervention with older adults- Lessons learned	Headley	2013	U.S.	Adults age 65 and over and self-reported being sedentary or involved in low to moderate physical activity programs	A gated community and a retirement community in mid-western region of the US	Quasi experimental	Quantitative	I	Y	D	Y	C	M	D	M	D	N	N	N	N	Physical activity
Translating the PLAYgrounds program into practice: A process evaluation using the RE-AIM framework	Janssen	2013	The Netherlands	Primary school children ages 6 to 12	Schools located west of Amsterdam in low SES area	RCT	Quantitative	I&S	Y	D	Y	D	Y	D	Y	D	N	N	Y	D	Physical activity
Reaching families at their homes for an intervention to reduce tobacco smoke exposure among infants	Kastirke	2013	Germany	Households with an infant age 3 years or younger in a rural region	Homes, rural region in the German Federal State of Mecklenburg-West Pomerania	Quasi-experimental	Quantitative	I	Y	D	N	N	N	N	N	N	N	N	N	N	Substance

Appendix D. Summary of all included RE-AIM studies (*N*=101)

Title	First Author	Year	Country	Target Population	Study Setting	Study Design	Methods Used	Level of Analysis	Reach Assessed? *	Reach Intervened? +	Effectiveness Assessed? *	Effectiveness Intervened? +	Adoption Assessed? *	Adoption Intervened? +	Implementation Assessed? *	Implementation Intervened? +	Maintenance(I)_Assessed? *	Maintenance(I)_Intervened? +	Maintenance(O)_Assessed? *	Maintenance(O)_Intervened? +	Outcome
Translation of an evidence-based asthma intervention: Physician Asthma Care Education (PACE) in the United States and Australia.	Patel	2013	USA & Australia	Physician-pediatricians and pediatric patients with asthma, parents	General medical practices	RCT/Observational/Translational	Mixed Methods	I&S	Y	D	Y	C	Y	C	Y	D	Y	C	N	N	No individual outcome
Ask-advise-connect: A new approach to smoking treatment delivery in health care settings	Vidrine	2013	U.S.	Current smokers 18 years or older who presented for care at any of the 10 clinics	10 family practice cities in urban area (Houston)	RCT	Quantitative	I	Y	D	Y	D	Y	D	Y	D	N	N	N	N	Substance
Promoting walking among office employees - evaluation of a randomized controlled intervention with pedometers and e-mail messages	Aittasalo	2012	Finland	Worksite employees	Occupational health care units	RCT	Quantitative	I	M	D	Y	C	Y	D	Y	D	Y	C	Y	D	Physical activity
Adoption, reach and effectiveness of computer-based, practitioner delivered and combined smoking interventions in general medical practices: A three-arm cluster randomized trial	Anesetti-Rothermel	2012	U.S.	Teenagers in West Virginia	Regional educational service agency	Evaluation	Quantitative	I	Y	D	Y	D	Y	D	Y	D	Y	D	Y	D	Substance

Appendix D. Summary of all included RE-AIM studies (*N*=101)

Title	First Author	Year	Country	Target Population	Study Setting	Study Design	Methods Used	Level of Analysis	Reach Assessed? *	Reach Intervened? +	Effectiveness Assessed? *	Effectiveness Intervened? +	Adoption Assessed? *	Adoption Intervened? +	Implementation Assessed? *	Implementation Intervened? +	Maintenance(I)_Assessed? *	Maintenance(I)_Intervened? +	Maintenance(O)_Assessed? *	Maintenance(O)_Intervened? +	Outcome
Applying the RE-AIM framework to evaluate two implementation strategies used to introduce a tool for lifestyle intervention in Swedish primary health care	Carlfjord	2012	Sweden	Providers in Swedish primary care settings	Swedish primary health care settings	Translational	Quantitative	S	M	D	M	C	M	C	Y	C	N	N	N	N	No individual outcome
Adoption, reach and effectiveness of computer-based, practitioner delivered and combined smoking interventions in general medical practices: A three-arm cluster randomized trial	Christian	2012	Germany	Adult smoking patients	General medical practices in north-east Germany	RCT	Quantitative	I&S	Y	C	Y	C	Y	C	Y	D	N	N	N	N	Substance
Motivational interviewing training to promote Head Start children's adherence to oral health care recommendations: Results of a program evaluation	Cook	2012	U.S.	Head Start children	Head Start schools	Pre-post	Quantitative	I	3	D	M	D	Y	D	Y	C	N	N	Y	D	Oral health care
State-wide dissemination of a school-based nutrition education programme: A RE-AIM (Reach, Efficacy, Adoption, Implementation, Maintenance) analysis	Dunton	2012	U.S.	3rd grade students & teachers	22 public elementary schools in California	RCT	Quantitative	I	Y	D	Y	D	Y	D	Y	D	Y	D	Y	D	No individual outcomes
Evaluating the implementation of a hospital work-site obesity prevention intervention: Applying the RE-AIM framework	Estabrook	2012	U.S.	Hospital workers	Worksite	RCT	Mixed Methods	S	Y	D	Y	C	Y	D	Y	D	M	D	Y	D	Multiple Outcomes

Appendix D. Summary of all included RE-AIM studies (*N*=101)

Title	First Author	Year	Country	Target Population	Study Setting	Study Design	Methods Used	Level of Analysis	Reach Assessed? *	Reach Intervened? +	Effectiveness Assessed? *	Effectiveness Intervened? +	Adoption Assessed? *	Adoption Intervened? +	Implementation Assessed? *	Implementation Intervened? +	Maintenance(I)_Assessed? *	Maintenance(I)_Intervened? +	Maintenance(O)_Assessed? *	Maintenance(O)_Intervened? +	Outcome
Calculating reach of evidence-based weight loss and memory improvement interventions among older adults attending Arkansas senior centers	Felix	2012	U.S.	Arkansas seniors	Senior centers	RCT	Quantitative	I	Y	D	N	N	N	N	N	N	N	N	N	N	Weight
Implementation outcomes of evidence-based quality improvement for depression in VA community based outpatient clinics	Fortney	2012	U.S.	Veterans	3 VA medical centers and their community-based 0utpatient clinics	Quasi experimental	Quantitative	I&S	Y	D	Y	C	Y	C	Y	C	N	N	Y	D	No individual outcome
12-month outcomes of an internet-based diabetes self-management support program	Glasgow	2012	U.S.	Adults	Primary care clinics	RCT	Quantitative	I	Y	D	Y	C	N	N	N	N	Y	B	N	N	Multiple outcomes
Processes and outcomes of developing a continuity of care document for use as a personal health record by people living with HIV/AIDS in New York City	Gordon	2012	U.S.	People living with HIV in New York City	New York Presbyterian System Select Health	Translational	Mixed Methods	I&S	Y	D	Y	C	Y	D	Y	C	N	N	Y	D	No individual outcome

Appendix D. Summary of all included RE-AIM studies (*N*=101)

Title	First Author	Year	Country	Target Population	Study Setting	Study Design	Methods Used	Level of Analysis	Reach Assessed? *	Reach Intervened? +	Effectiveness Assessed? *	Effectiveness Intervened? +	Adoption Assessed? *	Adoption Intervened? +	Implementation Assessed? *	Implementation Intervened? +	Maintenance(I)_Assessed? *	Maintenance(I)_Intervened? +	Maintenance(O)_Assessed? *	Maintenance(O)_Intervened? +	Outcome
Circles of Care: Development and initial evaluation of a peer support model for African-Americans with advanced cancer	Hanson	2012	U.S.	African-Americans with advanced cancer/serious illness living in three-county area of central North Carolina, Lay health volunteer advisors	Community (faith-based, cancer-support, community service organizations and African-American fraternities, sororities and local student health service clubs)	Observational evaluation	Mixed Methods	I&S	M	D	N	N	M	C	Y	D	N	N	N	N	No individual outcome
The GLAMA (Girls! Lead! Achieve! Mentor! Activate!) physical activity and peer leadership intervention pilot project: A process evaluation using the RE-AIM framework	Jenkison	2012	Australia	Teachers, school, and students (girls only)	One rural secondary school in Australia	Process evaluation	Mixed Methods	I&S	Y	D	M	C	Y	D	Y	C	N	N	N	N	Physical activity
Application of the RE-AIM framework to evaluate the impact of a worksite-based financial incentive intervention for smoking cessation	Kim	2012	U.S.	Adult smokers employed at General Electric worksites	Worksite	RCT & observational	Mixed Methods	I&S	Y	D	Y	D	Y	D	Y	D	N	N	Y	D	Substance

Appendix D. Summary of all included RE-AIM studies (*N*=101)

Title	First Author	Year	Country	Target Population	Study Setting	Study Design	Methods Used	Level of Analysis	Reach Assessed? *	Reach Intervened? +	Effectiveness Assessed? *	Effectiveness Intervened? +	Adoption Assessed? *	Adoption Intervened? +	Implementation Assessed? *	Implementation Intervened? +	Maintenance(I)_Assessed? *	Maintenance(I)_Intervened? +	Maintenance(O)_Assessed? *	Maintenance(O)_Intervened? +	Outcome
Evaluating the impact of patient's online access to doctor's visit notes: Designing and executing the OpenNotes project	Leveille	2012	U.S.	Primary care doctors and patients	Primary care practices in three diverse health systems Boston, MA, rural Northeastern PA and Seattle, WA	experimental, nonequivalent group pragmatic trial with pre-	Mixed Methods	I&S	Y	D	N	D	Y	D	N	D	N	D	N	N	No individual outcomes
Adoption, reach and effectiveness of computer-based, practitioner delivered and combined smoking interventions in general medical practices: A three-arm cluster randomized trial	Meyera	2012	Germany	Adult smoking patients	General medical practices	RCT	Quantitative	I&S	Y	C	Y	C	Y	C	Y	D	N	N	N	N	Substance
Successful dissemination of Fun 5 - a physical activity and nutrition program for children	Nigg	2012	U.S.	Elementary school children in after-school programs	Hawaii after-school settings serving elementary school-aged children	Dissemination	Mixed Methods	I&S	Y	D	Y	C	M	C	Y	D	N	N	Y	D	Multiple Outcomes
Differences in reach and attrition between web-based and print-delivered tailored interventions among adults over 50 years of age: Clustered randomized trial	Peels	2012	Netherlands	Adults > 50 yrs. of age	Communities in Municipal Health Council	RCT	Quantitative	I&S	Y	D	Y	D	Y	D	N	N	N	N	N	N	Other- Drop Out Rate

Appendix D. Summary of all included RE-AIM studies (*N*=101)

Title	First Author	Year	Country	Target Population	Study Setting	Study Design	Methods Used	Level of Analysis	Reach Assessed? *	Reach Intervened? +	Effectiveness Assessed? *	Effectiveness Intervened? +	Adoption Assessed? *	Adoption Intervened? +	Implementation Assessed? *	Implementation Intervened? +	Maintenance(I)_Assessed? *	Maintenance(I)_Intervened? +	Maintenance(O)_Assessed? *	Maintenance(O)_Intervened? +	Outcome
Rationale, design, and sample characteristics of a practical randomized trial to assess a weight loss intervention for low-income women: The Weight-Wise II Program	Samuel-Hodge	2012	U.S.	Mid-life low-income women between 40 & 64 years old	Community health departments in North Carolina	Translational	Mixed Methods	I&S	Y	D	N	N	Y	D	Y	D	N	N	N	N	Substance
Implementing an evidence-based caregiver intervention within an integrated healthcare system	Stevens	2012	U.S.	Dementia caregivers	Integrated healthcare system (1 hospital & 1 ambulatory internal medicine prim. care clinic) in Texas	Translational implementation	Quantitative	I&S	Y	D	Y	C	Y	D	Y	D	Y	D	Y	D	Caregiving
Adapting and RE-AIMing a heart disease prevention program for older women with diabetes	Toobert	2012	U.S.	Older Latina women w/ type 2 diabetes who were at risk for CHD, but do not have CHD; Viva Bien: high risk older Latina women who did not have CHD	Primary care practices in Oregon	RCT	Quantitative	I	Y	D	Y	C	Y	D	Y	D	Y	C	Y	C	Disease self-management

Appendix D. Summary of all included RE-AIM studies (*N*=101)

Title	First Author	Year	Country	Target Population	Study Setting	Study Design	Methods Used	Level of Analysis	Reach Assessed? *	Reach Intervened? +	Effectiveness Assessed? *	Effectiveness Intervened? +	Adoption Assessed? *	Adoption Intervened? +	Implementation Assessed? *	Implementation Intervened? +	Maintenance(I)_Assessed? *	Maintenance(I)_Intervened? +	Maintenance(O)_Assessed? *	Maintenance(O)_Intervened? +	Outcome
Implementation of an inpatient smoking cessation programme in a Veterans Affairs facility	Vick	2012	U.S.	Inpatient veteran smokers	General medical practices	RCT	Quantitative	I	Y	D	Y	N	Y	D	Y	D	N	N	N	N	Dietary
Baseline reach and adoption characteristics in a randomized controlled trial of two weight loss interventions translated into primary care: A structured report of real-world applicability	Yank	2012	U.S.	Overweight/ obese adults with increased cardio-metabolic risk	Primary care clinics	RCT	Quantitative	I	Y	C	N	N	Y	D	N	N	N	N	N	N	Diabetes
Exploring community gardens in a health disparate population: Findings from a Mixed Methods pilot study	Zoellner	2012	U.S.	Parents and youths from the Dan River Region	Community	Quasi-experimental	Mixed Methods	S	Y	D	Y	D	N	N	Y	N	Y	N	M	D	Diet
Improving prescribing safety in patients with renal insufficiency in the ambulatory setting: the Drug Renal Alert Pharmacy (DRAP) program	Bhardwaja	2011	U.S.	Integrated health care system	Population based; A large integrated health care delivery system	RCT	Quantitative	S	Y	D	Y	C	Y	D	Y	D	Y	D	Y	D	Medical adherence
Strategies to promote high school students' healthful food choices	Bukhari	2011	U.S.	High school students in Brooklyn, NY	High school	RCT	Mixed Methods	I	Y	D	Y	C	Y	D	Y	D	N	N	Y	D	Dietary

Appendix D. Summary of all included RE-AIM studies (*N*=101)

Title	First Author	Year	Country	Target Population	Study Setting	Study Design	Methods Used	Level of Analysis	Reach Assessed? *	Reach Intervened? +	Effectiveness Assessed? *	Effectiveness Intervened? +	Adoption Assessed? *	Adoption Intervened? +	Implementation Assessed? *	Implementation Intervened? +	Maintenance(I)_Assessed? *	Maintenance(I)_Intervened? +	Maintenance(O)_Assessed? *	Maintenance(O)_Intervened? +	Outcome
Engagement in a diabetes self-management website: usage patterns and generalizability of program use	Glasgow	2011	U.S.	Adults w/ T2DM	5 primary care clinics within Kaiser Permanente Colorado	randomized practical	Quantitative	I	Y	D	Y	C	N	N	Y	D	N	N	N	N	Multiple outcomes
RE-AIM evaluation of the Veterans Health Administration's MOVE! weight management program	Kahwati	2011	U.S.	VA patients	All VHA facilities	Translational	Mixed Methods	I&S	Y	C	Y	C	Y	C	Y	C	N	N	Y	C	Weight
Why are financial incentives not effective at influencing some smokers to quit? Results of a process evaluation of a worksite trial assessing the efficacy of financial incentives for smoking cessation	Kim	2011	U.S.	Adult employees at company work sites who smoked cigarettes	Community	RCT	Mixed Methods	I	N	N	Y	D	N	N	Y	D	N	N	N	N	Substance
Understanding dissemination and implementation of a new intervention in assisted living settings: The case of function-focused care	Resnick	2011	U.S.	Residents of assisted living	Assisted living communities	RCT	Mixed Methods	S	M	D	Y	C	Y	D	Y	D	N	N	Y	B	Physical activity
Intervention costs and cost-effectiveness for a multiple-risk-factor diabetes self-management trial for Latinas: economic analysis of ¡Viva Bien!	Ritzwoller	2011	U.S.	Latinas with T2DM	Primary care clinics	RCT	Quantitative	I	Y	D	Y	C	N	D	N	N	N	N	N	N	Multiple outcomes

Appendix D. Summary of all included RE-AIM studies (*N*=101)

Title	First Author	Year	Country	Target Population	Study Setting	Study Design	Methods Used	Level of Analysis	Reach Assessed? *	Reach Intervened? +	Effectiveness Assessed? *	Effectiveness Intervened? +	Adoption Assessed? *	Adoption Intervened? +	Implementation Assessed? *	Implementation Intervened? +	Maintenance(I)_Assessed? *	Maintenance(I)_Intervened? +	Maintenance(O)_Assessed? *	Maintenance(O)_Intervened? +	Outcome
RE-AIM evaluation of the alcohol and pregnancy project: Educational resources to inform health professionals about prenatal alcohol exposure and fetal alcohol spectrum disorder	Payne	2011	Australia	Health professionals in Western Australia	Dept. of Health, Aboriginal Community Controlled Health Services, & the Australia Medical Association	Quasi-experimental w/o control group	Quantitative	I	M	D	Y	C	Y	D	Y	D	Y	D	Y	D	No individual outcome
The impact of disseminating the whole-community project '10,000 Steps': a RE-AIM analysis	Van Acker	2011	Belgium	Adult citizens living in the Flanders region of Belgium	Community-based	Evaluation of adaptations	Mixed Methods	I	Y	D	Y	D	Y	D	Y	D	N	N	Y	D	Physical Activity
The long-term efficacy of two computer-tailored physical activity interventions for older adults: Main effects and mediators	van Stralen	2011	Netherlands	Adults 50 years and older	Municipal health council region communities in the Netherlands	RCT	Quantitative	I&S	Y	D	Y	C	Y	D	Y	D	Y	D	N	N	Physical Activity
Lay health educators translate a weight-loss intervention in senior centers: a randomized controlled trial	West	2011	U.S.	Seniors	Seniors' centers	RCT	Quantitative	S	Y	D	Y	C	Y	D	Y	D	N	N	N	N	Weight
H.U.B city steps: methods and early findings from a community-based participatory research trial to reduce blood pressure among African Americans	Zoellner	2011	U.S.	African American adults in low-income Southern state	Community	Quasi-experimental	Mixed Methods	I	Y	C	Y	C	Y	D	Y	D	N	N	N	N	Quality of life

Appendix D. Summary of all included RE-AIM studies (*N*=101)

Title	First Author	Year	Country	Target Population	Study Setting	Study Design	Methods Used	Level of Analysis	Reach Assessed? *	Reach Intervened? +	Effectiveness Assessed? *	Effectiveness Intervened? +	Adoption Assessed? *	Adoption Intervened? +	Implementation Assessed? *	Implementation Intervened? +	Maintenance(I)_Assessed? *	Maintenance(I)_Intervened? +	Maintenance(O)_Assessed? *	Maintenance(O)_Intervened? +	Outcome
Evaluating an insurance-sponsored weight management program with the RE-AIM model, West Virginia, 2004-2008	Abildso	2010	U.S.	Obese adults members covered by the insurance agency	31 fitness facilities: 2 in university cardiac rehab/ exercise labs, 2 in physical therapy facilities, 5 in hospitals & 22 private wellness/ fitness centers	Program evaluation	Quantitative	I&S	Y	D	Y	C	Y	D	Y	D	Y	C	Y	D	Multiple outcomes
From research to practice: EnhanceFitness, an innovative community-based senior exercise program.	Belza	2010	U.S.	Older adults	Any organization that provides senior services - program is currently in 19 states and DC	Dissemination	Quantitative	S	Y	D	Y	C	Y	D	Y	D	N	N	Y	D	Physical activity
The WellingTONNE challenge toolkit: Using the RE-AIM framework to evaluate a community resource promoting healthy lifestyle behaviours.	Caperchione	2010	Australia	Applicant organizations	Community organizations	Process evaluation	Quantitative	I&S	Y	D	Y	N	Y	D	Y	C	Y	N	Y	D	Multiple outcomes
Process evaluation of a school based physical activity related injury prevention programme using the RE-AIM framework	Collard	2010	Netherlands	Children aged 10-12 years in Dutch primary schools	Netherland elementary schools	Process evaluation of RCT	Qualitative	I&S	Y	D	N	C	Y	D	Y	D	Y	D	N	N	Physical activity

Appendix D. Summary of all included RE-AIM studies (*N*=101)

Title	First Author	Year	Country	Target Population	Study Setting	Study Design	Methods Used	Level of Analysis	Reach Assessed? *	Reach Intervened? +	Effectiveness Assessed? *	Effectiveness Intervened? +	Adoption Assessed? *	Adoption Intervened? +	Implementation Assessed? *	Implementation Intervened? +	Maintenance(I)_Assessed? *	Maintenance(I)_Intervened? +	Maintenance(O)_Assessed? *	Maintenance(O)_Intervened? +	Outcome
Translation of a dementia caregiver intervention for delivery in homecare as a reimbursable medicare service: Outcomes and lessons learned	Gitlin	2010	U.S.	Families of dementia patients	Fox physical therapy service in 5 mid Atlantic states	Evaluation	Quantitative	I&S	Y	D	Y	D	Y	D	Y	D	Y	D	N	N	No individual outcome
Recruitment for an Internet-based diabetes self-management program: Scientific and ethical implications	Glasgow	2010	U.S.	T2DM patients at Kaiser Permanente - Colorado	Outside primary care setting by research staff members	RCT	Quantitative	I	Y	D	N	N	Y	D	N	N	N	N	N	N	Multiple outcomes
Outcomes of minimal and moderate support versions of an internet-based diabetes self-management support program	Glasgow	2010	U.S.	Adults	Clinical	RCT	Quantitative	I	Y	D	Y	C	N	N	Y	D	N	N	N	N	Multiple outcomes
Participation in an ambulatory e-pharmacovigilance system	Haas	2010	U.S.	Patients receiving a prescription for a target medication	Interactive Voice Response System	Cross-sectional	Quantitative	I	Y	D	N	N	N	N	N	N	N	N	N	N	Substance
Implementation of a pilot primary care lifestyle change intervention for families of pre-school children: Lessons learned	McKee	2010	U.S.	Families of pre-school children	Urban practices (health centers)	RCT	Mixed Methods	I&S	Y	D	Y	B	Y	D	Y	D	N	N	N	N	Multiple outcomes

Appendix D. Summary of all included RE-AIM studies (*N*=101)

**Title**	**First Author**	**Year**	**Country**	**Target Population**	**Study Setting**	**Study Design**	**Methods Used**	**Level of Analysis**	**Reach Assessed? ***	**Reach Intervened? +**	**Effectiveness Assessed? ***	**Effectiveness Intervened? +**	**Adoption Assessed? ***	**Adoption Intervened? +**	**Implementation Assessed? ***	**Implementation Intervened? +**	**Maintenance(I)_Assessed? ***	**Maintenance(I)_Intervened? +**	**Maintenance(O)_Assessed? ***	**Maintenance(O)_Intervened? +**	**Outcome**
Optimized probability sampling of study sites to improve generalizability in a multisite intervention trial	Kraschnewski	2010	U.S.	Low-income women	NC health departments	preparation phase 1 of 2-	Mixed Methods	S	N	N	N	N	Y	D	N	N	N	N	N	N	Substance
Step Ahead: A worksite obesity preventive trial among hospital employees	Lemon	2010	U.S.	Hospital workers	Worksite	RCT	Quantitative	I	Y	D	Y	C	Y	C	Y	D	N	N	N	N	Multiple Outcomes
Smoking cessation for hospitalized smokers: an evaluation of the "Ottawa Model"	Reid	2010	Canada	Hospitalized smokers	Hospitals; small rural community hospitals to large urban academic teaching centers	Quasi-experimental	Mixed Methods	I	Y	D	Y	D	Y	D	Y	D	N	N	N	N	Substance
Coaches' perspectives on implementing an evidence-informed injury prevention programme in junior community netball	Saunders	2010	Australia	Coaches of junior community netball teams	Community	Observational / translational	Mixed Methods	I	Y	D	Y	B	Y	D	Y	D	Y	D	N	N	No behavioral outcomes

Appendix D. Summary of all included RE-AIM studies (*N*=101)

Title	First Author	Year	Country	Target Population	Study Setting	Study Design	Methods Used	Level of Analysis	Reach Assessed? *	Reach Intervened? +	Effectiveness Assessed? *	Effectiveness Intervened? +	Adoption Assessed? *	Adoption Intervened? +	Implementation Assessed? *	Implementation Intervened? +	Maintenance(I)_Assessed? *	Maintenance(I)_Intervened? +	Maintenance(O)_Assessed? *	Maintenance(O)_Intervened? +	Outcome
Seven-year follow-up of a multiple-health behavior diabetes intervention-HMD	Toobert	2010	U.S.	Post-menopausal women with T2DM at risk for CHD	Primary care clinics	RCT	Quantitative	I	Y	D	Y	D	Y	D	N	N	Y	D	N	N	Multiple outcomes
¡Viva Bien!: Overcoming recruitment challenges in a multiple-risk-factor diabetes trial	Toobert	2010	U.S.	Latinas with T2DM	19 health clinics	RCT	Quantitative	I	Y	D	N	N	Y	N	N	N	N	N	N	N	Multiple outcomes
Exploring the public health impact of an intensive exercise program for patients with rheumatoid arthritis: A dissemination and implementation study	Van der Giesen	2010	Netherlands	Rheumatoid arthritis patients	Does not explicitly state; assuming each rhuemotolist's office	RCT	Quantitative	I	Y	D	Y	D	Y	D	Y	D	N	D	Y	D	Disease self-management

Appendix D. Summary of all included RE-AIM studies (*N*=101)

Title	First Author	Year	Country	Target Population	Study Setting	Study Design	Methods Used	Level of Analysis	Reach Assessed? *	Reach Intervened? +	Effectiveness Assessed? *	Effectiveness Intervened? +	Adoption Assessed? *	Adoption Intervened? +	Implementation Assessed? *	Implementation Intervened? +	Maintenance(I)_Assessed? *	Maintenance(I)_Intervened? +	Maintenance(O)_Assessed? *	Maintenance(O)_Intervened? +	Outcome
A preliminary report of knowledge translation: Lessons from taking screening and brief intervention techniques from the research setting into regional systems of care	Bernstein	2009	U.S.	Adults in 7 emergency departments (EDs) in Massachusetts	Massachusetts in 7 of 74 EDs using health promotion advocates	Translational	Mixed Methods	S	M	D	M	D	M	D	M	D	N	N	Y	D	No individual outcomes
Using RE-AIM framework to evaluate the statewide dissemination of a school-based physical activity and nutrition curriculum 'exercise your options'	Dunton	2009	U.S.	Statewide (California) middle school children	Middle schools in California	Evaluation	Quantitative	I&S	Y	D	Y	C	Y	D	Y	D	Y	D	N	N	Multiple outcomes
Reach and effectiveness of DVD and in-person diabetes self-management education	Glasgow	2009	U.S.	Adults	Clinical	RCT	Quantitative	I	Y	D	Y	C	N	N	Y	D	N	N	N	N	Multiple outcomes
ParticipACTION: Baseline assessment of the 'new ParticipACTION': A quantitative survey of Canadian organizational awareness and capacity	Plotnikoff	2009	Canada	Organizations	Organizations involved in physical activity and health promotion	Observational Cross-sectional	Quantitative	S	Y	D	N	N	Y	D	Y	D	N	N	N	N	No individual outcomes
A randomized, controlled trial of financial incentives for smoking cessation	Volpp	2009	U.S.	Adult employees at company work sites who smoked cigarettes	Community	RCT	Quantitative	I	Y	D	Y	C	N	N	N	N	N	N	N	N	Substance

Appendix D. Summary of all included RE-AIM studies (*N*=101)

Title	First Author	Year	Country	Target Population	Study Setting	Study Design	Methods Used	Level of Analysis	Reach Assessed? *	Reach Intervened? +	Effectiveness Assessed? *	Effectiveness Intervened? +	Adoption Assessed? *	Adoption Intervened? +	Implementation Assessed? *	Implementation Intervened? +	Maintenance(I)_Assessed? *	Maintenance(I)_Intervened? +	Maintenance(O)_Assessed? *	Maintenance(O)_Intervened? +	Outcome
Determining the impact of Walk Kansas: Applying a team-building approach to community physical activity promotion	Estabrooks	2008	U.S.	Citizens in Kansas	Kansas	Pre-post	Quantitative	I	Y	D	Y	C	Y	D	N	N	Y	C	Y	D	Physical activity
Evaluating initial reach and robustness of a practical randomized trial of smoking reduction	Glasgow	2008	U.S.	Smokers scheduled for outpatient surgery or an invasive medical procedure	Clinical	RCT	Quantitative	I	Y	D	Y	C	N	N	Y	D	N	N	N	N	Multiple outcomes
The community health educator referral liaison (CHERL): A primary care practice role for promoting healthy behaviors	Holtrop	2008	U.S.	Adult patients with unhealthy behaviors	Primary care practice	RCT	Quantitative	I	Y	D	Y	C	Y	D	Y	D	N	N	Y	D	Multiple outcomes
Translation of effective tai chi intervention into a community-based falls-prevention program	Li	2008	U.S.	Community-living older adults	Community-based senior service providers	Pre-post	Quantitative	I	Y	D	Y	C	Y	D	Y	D	Y	D	Y	D	Multiple outcomes
Impact of a print intervention to increase annual mammography screening among Korean American women enrolled in the National Breast and Cervical Cancer Early Detection Program	Maxwell	2008	U.S.	Korean-American low-income women	Home-based	Quasi-experimental	Mixed Methods	I	Y	D	Y	D	Y	D	Y	D	N	N	Y	D	Disease self-management

Appendix D. Summary of all included RE-AIM studies (*N*=101)

Title	First Author	Year	Country	Target Population	Study Setting	Study Design	Methods Used	Level of Analysis	Reach Assessed? *	Reach Intervened? +	Effectiveness Assessed? *	Effectiveness Intervened? +	Adoption Assessed? *	Adoption Intervened? +	Implementation Assessed? *	Implementation Intervened? +	Maintenance(I)_Assessed? *	Maintenance(I)_Intervened? +	Maintenance(O)_Assessed? *	Maintenance(O)_Intervened? +	Outcome
From innovation to practices: initiation, implementation and evaluation of a physician-based physical activity promotion programme in Finland	Aittasalo	2007	Finland	Municipal health centers and occupational health care physicians in Finland	Municipal health centers, occupational health care, private clinics and rehab centers, hospitals	Evaluation	Quantitative	I&S	Y	D	Y	C	Y	C	Y	D	N	N	Y	D	No individual outcomes
Evaluating active U: An internet-mediated physical activity program	Buis	2007	U.S.	Faculty, staff, graduate students at University of Michigan during winter months	University of Michigan	Evaluation	Quantitative	I	Y	D	M	C	M	D	Y	D	N	N	3	D	No individual outcomes
Using the RE-AIM framework to evaluate a physical activity intervention in churches	Bopp	2007	U.S.	African Methodist churches across South Carolina	African Methodist Episcopal Churches across South Carolina	Evaluation	Mixed Methods	I&S	Y	D	Y	C	M	D	Y	D	Y	C	Y	C	Physical activity
Process evaluation of an effective church-based diet intervention: Body & soul	Campbell	2007	U.S.	African-American churches	Churches	RCT	Mixed Methods	S	Y	D	Y	C	Y	D	Y	D	N	N	Y	D	Multiple outcomes

Appendix D. Summary of all included RE-AIM studies (*N*=101)

Title	First Author	Year	Country	Target Population	Study Setting	Study Design	Methods Used	Level of Analysis	Reach Assessed? *	Reach Intervened? +	Effectiveness Assessed? *	Effectiveness Intervened? +	Adoption Assessed? *	Adoption Intervened? +	Implementation Assessed? *	Implementation Intervened? +	Maintenance(I)_Assessed? *	Maintenance(I)_Intervened? +	Maintenance(O)_Assessed? *	Maintenance(O)_Intervened? +	Outcome
Resources for health: A primary care based diet and physical activity intervention targeting urban Latinos with multiple chronic conditions	Eakin	2007	U.S.	Low income Spanish-speaking people with chronic conditions	Clinica Campesina Family Health Services, a community health center in north urban Denver	RCT	Quantitative	I	Y	D	Y	C	Y	D	Y	D	N	N	N	N	Multiple outcomes
Innovation in aged care: Well for Life: Evaluation and policy implications of a health promotion initiative for frail older people in aged care settings.	McKenzie	2007	Australia	Older adults in aged care settings	Planned activity groups and residential aged care facilities	Observational /Translational?	Mixed Methods	I&S	Y	D	M	D	Y	D	Y	D	N	N	N	N	No individual outcomes
Beyond effectiveness: Evaluating the public health impact of the WISEWOMAN program	Farris	2007	U.S.	Midlife uninsured women in US	14 project sites across North Carolina	Evaluation	Quantitative	S	Y	D	Y	C	N	N	N	N	Y	C	N	N	Other- Blood Pressure
Long-term effects of the Mediterranean lifestyle program: A randomized clinical trial for postmenopausal women with type 2 diabetes	Toobert	2007	U.S.	Post-menopausal women with T2DM	Retreat/ Primary care clinics	RCT	Quantitative	I	Y	D	Y	D	Y	D	Y	D	Y	C	Y	D	Multiple outcomes

Appendix D. Summary of all included RE-AIM studies (*N*=101)

Title	First Author	Year	Country	Target Population	Study Setting	Study Design	Methods Used	Level of Analysis	Reach Assessed? *	Reach Intervened? +	Effectiveness Assessed? *	Effectiveness Intervened? +	Adoption Assessed? *	Adoption Intervened? +	Implementation Assessed? *	Implementation Intervened? +	Maintenance(I)_Assessed? *	Maintenance(I)_Intervened? +	Maintenance(O)_Assessed? *	Maintenance(O)_Intervened? +	Outcome
Effects of a brief computer-assisted diabetes self-management intervention on dietary, biological and quality of life outcomes	Glasgow	2006	U.S.	Adults	Clinical	RCT	Quantitative	I	Y	D	Y	C	N	N	Y	D	N	N	N	N	Multiple outcomes
Robustness of a computer-assisted diabetes self-management intervention across patient characteristics, healthcare settings and intervention staff	Glasgow	2006	U.S.	T2DM patients in Denver metro area	Outside primary care setting by research staff members	RCT	Quantitative	I	Y	D	Y	C	Y	D	Y	D	Y	D	N	N	Multiple outcomes
Partial examination of the public health impact of the people with arthritis can exercise (PACE) program: Reach, adoption, and maintenance	Gyrucik	2006	Canada	Participants on instructor-training workshops (PACE)	Those beginning a PACEs program	Cross-sectional	Mixed Methods	S	M	D	N	N	M	D	N	N	N	N	M	D	Multiple outcomes
Adverse outcomes in surgical patients: Implementation of a nationwide reporting system	Marang-van de Mheen	2006	The Netherlands	Surgeons and surgical residents in the Netherlands	Surgery departments in the Netherlands not including centers for children and private clinics	Cross-sectional	Quantitative	I&S	Y	D	Y	D	Y	D	Y	D	N	N	Y	D	Adherence to reporting surgical procedures

Appendix D. Summary of all included RE-AIM studies (*N*=101)

Title	First Author	Year	Country	Target Population	Study Setting	Study Design	Methods Used	Level of Analysis	Reach Assessed? *	Reach Intervened? +	Effectiveness Assessed? *	Effectiveness Intervened? +	Adoption Assessed? *	Adoption Intervened? +	Implementation Assessed? *	Implementation Intervened? +	Maintenance(I)_Assessed? *	Maintenance(I)_Intervened? +	Maintenance(O)_Assessed? *	Maintenance(O)_Intervened? +	Outcome
Economic analysis of the Mediterranean lifestyle program for postmenopausal women with diabetes	Ritzwoller	2006	U.S.	Post-menopausal women with diabetes at risk for CHD	Not reported	RCT	Quantitative	I	Y	D	Y	D	Y	D	N	N	N	N	N	N	Multiple outcomes
Translational research principles of an effectiveness trial for diabetes care in an urban African-American population.	Gary	2005	U.S.	Urban African American population	African Americans in the Baltimore MD urban area	RCT	Mixed Methods	I	Y	D	Y	C	Y	D	Y	C	Y	D	Y	D	No individual outcome
Randomized effectiveness trial of a computer-assisted intervention to improve diabetes care	Glasgow	2005	U.S.	_Patients with T2DM	Primary care offices	RCT	Quantitative	I	Y	D	Y	C	Y	D	Y	D	Y	C	N	N	Multiple outcomes
Randomized effectiveness trial of a computer-assisted intervention to improve diabetes care	Glasgow	2005	U.S.	Patients with T2DM	Primary care offices	RCT	Quantitative	I	Y	D	Y	C	Y	D	Y	D	Y	C	N	N	Multiple outcomes
Effects of the Mediterranean lifestyle program on multiple risk behaviors and psychosocial outcomes among women at risk for heart disease	Toobert	2005	U.S.	Post-menopausal women with T2DM who are at elevated risk for CHD	Primary care clinics and retreat	RCT	Quantitative	I	Y	D	Y	C	Y	D	N	N	N	N	N	N	Multiple outcomes

Appendix D. Summary of all included RE-AIM studies (*N*=101)

Title	First Author	Year	Country	Target Population	Study Setting	Study Design	Methods Used	Level of Analysis	Reach Assessed? *	Reach Intervened? +	Effectiveness Assessed? *	Effectiveness Intervened? +	Adoption Assessed? *	Adoption Intervened? +	Implementation Assessed? *	Implementation Intervened? +	Maintenance(I)_Assessed? *	Maintenance(I)_Intervened? +	Maintenance(O)_Assessed? *	Maintenance(O)_Intervened? +	Outcome
Physical activity promotion in primary care: Bridging the gap between research and practice	Eakin	2004	Australia	General practitioners	Clinics in Rockhampton, Australia	Evaluation	Quantitative	I&S	N	N	Y	D	Y	D	Y	D	Y	D	N	N	Physical activity
A practical randomized trial to improve diabetes care	Glasgow	2004	U.S.	Adult T2DM patients from primary care	Primary care offices	RCT	Quantitative	I	Y	D	Y	C	Y	D	Y	D	N	N	N	N	Multiple outcomes
Adoption, reach, and implementation of a novel smoking control program: Analysis of a public utility-research organization partnership	Glasgow	2004	U.S.	Smokers living within 50 miles of Eugene, OR	Home	RCT	Quantitative	I	Y	D	N	N	Y	D	Y	D	N	N	Y	C	Multiple outcomes
The D-Net diabetes self-management program: Long-term implementation, outcomes, and generalization results	Glasgow	2003	U.S.	Adult T2DM patients from participating primary care offices	Internet-based	RCT	Quantitative	I	N	N	N	N	N	N	Y	D	Y	C	N	N	Multiple outcomes
Biologic and quality-of-life outcomes from the Mediterranean lifestyle program: A randomized clinical trial	Toobert	2003	U.S.	_Post-menopausal women with T2DM at risk for CHD	Retreat/primary care clinics	RCT	Quantitative	I	Y	D	Y	C	N	N	N	N	N	N	N	N	Multiple outcomes

Appendix D. Summary of all included RE-AIM studies (*N*=101)

Title	First Author	Year	Country	Target Population	Study Setting	Study Design	Methods Used	Level of Analysis	Reach Assessed? *	Reach Intervened? +	Effectiveness Assessed? *	Effectiveness Intervened? +	Adoption Assessed? *	Adoption Intervened? +	Implementation Assessed? *	Implementation Intervened? +	Maintenance(I)_Assessed? *	Maintenance(I)_Intervened? +	Maintenance(O)_Assessed? *	Maintenance(O)_Intervened? +	Outcome
Implementation, generalization and long-term results of the "choosing well" diabetes self-management intervention	Glasgow	2002	U.S.	Adults 40 years of age and older with T2DM living near Eugene, OR	Clinical	RCT	Quantitative	I	N	N	Y	C	N	N	Y	D	N	N	Y	C	Multiple outcomes
If you build it, will they come? Reach and adoption associated with a comprehensive lifestyle management program for women with type 2 diabetes	Toobert	2002	U.S.	Postmenopausal women with type 2 diabetes who are at elevated risk for coronary heart disease (CHD).	Primary care clinics; Internet	RCT	Quantitative	I	Y	D	N	N	Y	D	N	N	N	N	Y	N	Multiple outcomes
Promoting smoking abstinence in pregnant and postpartum patients: A comparison of two approaches	Lando	2001	U.S.	Pregnant women who either smoked currently or had quit recently	Clinical	RCT	Quantitative	I	Y	D	N	N	Y	D	Y	D	N	N	Y	D	Substance
Brief, computer-assisted diabetes dietary self-management counseling: Effects on behavior, physiologic outcomes, and quality of life	Glasgow	2000	U.S.	Adults with T2DM 40 years and older	Community health center	RCT	Quantitative	I	Y	D	Y	C	Y	D	Y	D	N	N	N	N	Multiple outcomes
A brief smoking cessation intervention for women in low-income planned parenthood clinics	Glasgow	2000	U.S.	Women aged 15 to 35 who attend Planned Parenthood clinics for non-pregnancy related visits	4 Planned Parenthood clinics in Portland, OR	RCT	Quantitative	I	Y	D	Y	C	Y	D	Y	D	Y	C	N	N	Multiple outcomes

Appendix D. Summary of all included RE-AIM studies (*N*=101)

Title	First Author	Year	Country	Target Population	Study Setting	Study Design	Methods Used	Level of Analysis	Reach Assessed? *	Reach Intervened? +	Effectiveness Assessed? *	Effectiveness Intervened? +	Adoption Assessed? *	Adoption Intervened? +	Implementation Assessed? *	Implementation Intervened? +	Maintenance(I)_Assessed? *	Maintenance(I)_Intervened? +	Maintenance(O)_Assessed? *	Maintenance(O)_Intervened? +	Outcome
Reaching those most in need: Participation in a Planned Parenthood smoking cessation program	Eakin	1999	U.S.		3 Planned Parenthood clinics in the Portland, Oregon metropolitan area	RCT	Quantitative	I	Y	D	N	N	N	N	N	N	N	N	N	N	Substance

Level of Analysis: I= Individual; S= Setting; I&S= Combination of Individual and Setting; IWS= Individuals Clustered within Setting; * Assessment Variables: N= Not reported; Y= Accurately reported; M= Misreported; + Intervening Variables: N= Dimension not included in article; D= Described; C= Changed; B= Described and Changed
